# Toward mechanistic medical digital twins: some use cases in immunology

**DOI:** 10.3389/fdgth.2024.1349595

**Published:** 2024-03-07

**Authors:** Reinhard Laubenbacher, Fred Adler, Gary An, Filippo Castiglione, Stephen Eubank, Luis L. Fonseca, James Glazier, Tomas Helikar, Marti Jett-Tilton, Denise Kirschner, Paul Macklin, Borna Mehrad, Beth Moore, Virginia Pasour, Ilya Shmulevich, Amber Smith, Isabel Voigt, Thomas E. Yankeelov, Tjalf Ziemssen

**Affiliations:** ^1^ Department of Medicine, University of Florida, Gainesville, FL, United States; ^2^ Department of Mathematics and School of Biological Sciences, University of Utah, Salt Lake, UT, United States; ^3^ Department of Surgery, University of Vermont, Burlington, VT, United States; ^4^ Biotechnology Research Center, Technology Innovation Institute, Abu Dhabi, United Arab Emirates; ^5^ Biocomplexity Institute and Initiative, University of Virginia, Charlottesville, VA, United States; ^6^ Department of Intelligent Systems Engineering, Indiana University, Bloomington, IN, United States; ^7^ Department of Biochemistry, University of Nebraska, Lincoln, NE, United States; ^8^ U.S. Walter Reed Army Institute of Research, Silver Spring, MD, United States; ^9^ Department of Microbiology and Immunology, University of Michigan, Ann Arbor, MI, United States; ^10^ U.S. Army Research Office, Research Triangle Park, NC, United States; ^11^ Institute for Systems Biology, Seattle, WA, United States; ^12^ Department of Pediatrics, University of Tennessee Health Science Center, Memphis, TN, United States; ^13^ Center for Clinical Neuroscience, Carl Gustav Carus University Hospital, Dresden, Germany; ^14^ Department of Biomedical Engineering, Oden Institute for Computational Engineering and Sciences, Austin, TX, United States; ^15^ Departments of Biomedical Engineering, Diagnostic Medicine, Oncology, The University of Texas, Austin, TX, United States; ^16^ Department of Imaging Physics, The University of Texas MD Anderson Cancer Center, Austin, TX, United States

**Keywords:** medical digital twin, immune digital twin, personalized medicine, roadmap, review of digital twin projects

## Abstract

A fundamental challenge for personalized medicine is to capture enough of the complexity of an individual patient to determine an optimal way to keep them healthy or restore their health. This will require personalized computational models of sufficient resolution and with enough mechanistic information to provide actionable information to the clinician. Such personalized models are increasingly referred to as medical digital twins. Digital twin technology for health applications is still in its infancy, and extensive research and development is required. This article focuses on several projects in different stages of development that can lead to specific—and practical–medical digital twins or digital twin modeling platforms. It emerged from a two-day forum on problems related to medical digital twins, particularly those involving an immune system component. Open access video recordings of the forum discussions are available.

## Introduction

**Figure 1:**
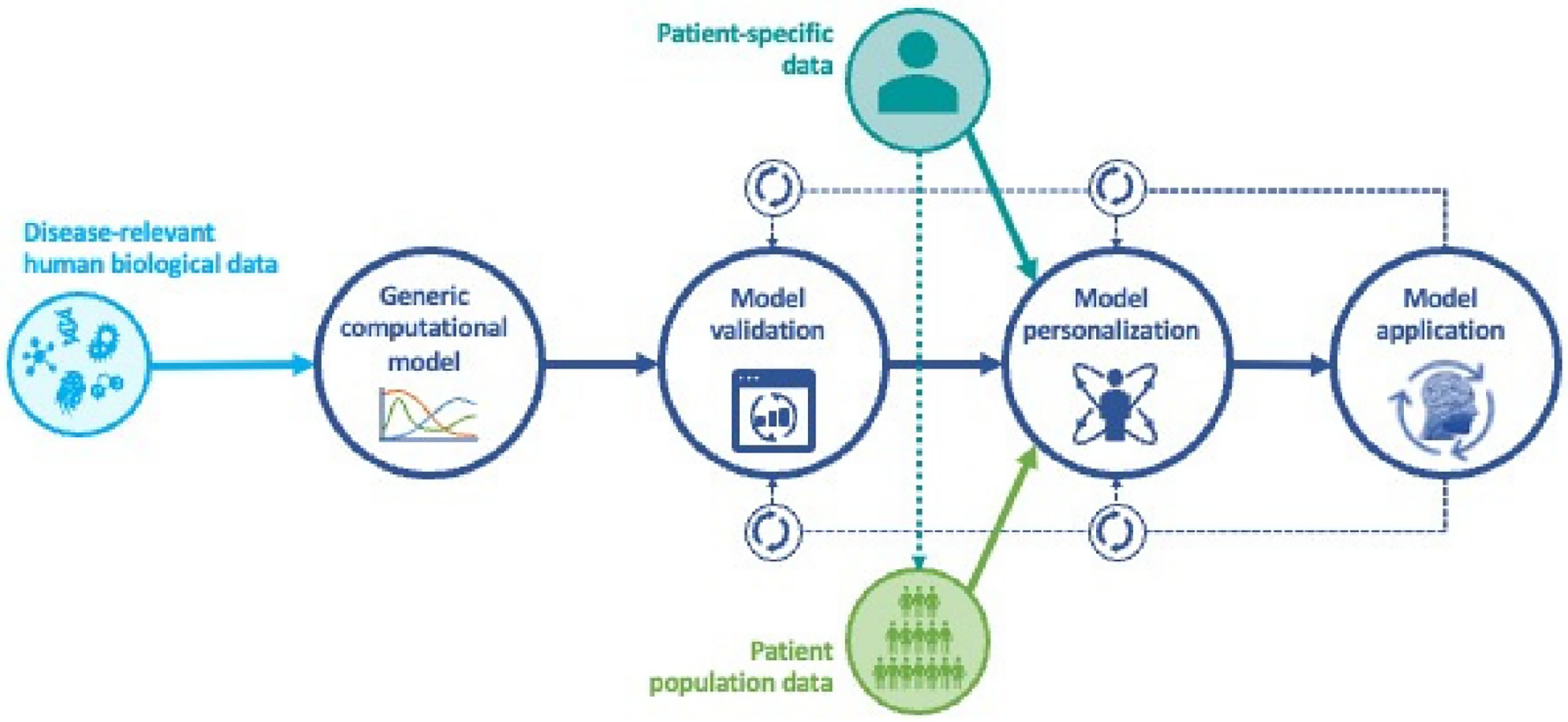
Workflow for development of medical digital twins.

The vision at the heart of personalized medicine is to design and implement customized approaches to keep individuals healthy and how to restore their health when it is compromised. This requires that we can quantify the differences between individuals that account for their health status in relation to their biological makeup, as well as the cumulative influences they are subjected to in their environment. Furthermore, we must also quantify their individual response to any therapeutic interventions over time. The only systematic way to accomplish this is to use computational models that can be personalized to an individual patient and can be dynamically recalibrated to reflect changes over time. Such models have become known as medical digital twins (MDTs). See Figure 1 for a workflow schematic of medical digital twin development. If we want to predict the individual response to treatment or develop novel drugs and other interventions, then these models need to be able to capture mechanisms and the effects of perturbing them. Given their use in patient treatment, requirements for model validation are much more stringent than for models used to discover new biology. See (1) for a detailed discussion of these requirements in the context of cancer modeling, but applicable more generally.

If we accept the hypothesis that the human body is a complex system, in the technical sense that very small perturbations can lead to very large effects on global system dynamics (the proverbial “butterfly effect”), then these computational models might need to be highly granular, depending on the nature of the interventions. Available data and model complexity will impose a lower limit on the resolution achievable. At the same time, many currently available therapeutic interventions cannot make use of highly granular patient-specific information. This puts limits on the utility of resolution above a certain limit. Thus, the construction of MDTs becomes a multi-objective optimization process: *build the lowest-resolution computational model of human biology for which patient data are available and that is sufficient to simulate available interventions*.

The use of digital twins in industry, where the concept originated [see (2) for a review of the concept's origins], is quite advanced, with broad applications for preventive maintenance, control, and design. The models used to capture machinery, physical plants, or production and management operations are almost entirely mechanistic, and the “Internet of Things” has made the data coupling of the physical part of a twin with the underlying model, its digital part, almost routine in many applications. In medicine, however, the state-of-the-art is considerably less advanced. While the industrial application of the digital twin concept is instructive, it differs from medical digital twins in several key aspects. Most importantly, human biology is not the result of a planned design, but the outcome of an evolutionary process, with many emergent properties. We do not have a complete theoretical understanding of biological systems, providing a list of general principles that could form the basis of computational models, as we do for physical systems. Two other characteristic features of biological systems are genotypic and phenotypic heterogeneity across individuals and stochasticity in system dynamics. All these features present massive challenges to mathematical modeling of individuals and populations.

Biological mechanisms cross scales as do therapeutic interventions. For instance, many drugs target intracellular mechanisms but have tissue- or organ-level effects. Therefore, many mechanistic MDTs will need to span multiple scales. This raises the question of whether our current repertoire of modeling paradigms is sufficient to form the basis of digital twins across various health conditions and how to choose the right type of model for each one. How can we effectively and credibly capture key features of human biology in a manner suitable for a specific clinical application while considering the diversity of patients and their individual characteristics? For many applications today, we have insufficient knowledge of the underlying human biology to contemplate building mechanistic MDTs and need to settle for patient stratification algorithms based on machine learning. In the relatively rare cases of sufficiently large patient data sets, models based on artificial intelligence approaches can be used.

To discuss these issues in depth, an event was held in Lake Nona, FL, February 23–24, 2023, the “Forum on Precision Immunology: Immune Digital Twins” (3), supported by the ARO Biomathematics Program. To focus the discussions, the topic was limited to the immune system, a key player in many of the most prevalent diseases humankind faces today, from infectious diseases to diabetes, cancer, autoimmune and heart disease. Furthermore, the discussion was limited largely to the development and use of MDTs with underlying mathematical models that incorporate known biological mechanisms and have been calibrated and validated with experimental evidence. One aim was to assess examples of ongoing modeling projects that are part of MDT development related to immunity. The focus of this article is to describe some of these projects, at different stages of development toward viable MDT technology. Video recordings of the presentations and discussions are available at (3) (presentation titles on the webpage are hyperlinks). Parts of this paper are included in the preprint (4) by the same set of authors.

There are many important issues that were not discussed at the Forum and are not addressed in this article, due to lack of time and required expertise among the participants. First, there are several sources of uncertainty in MDT predictions, including uncertainty about the model used, about the data used, patient heterogeneity, to name the most important ones. Second, there is a plethora of privacy, legal, and ethical issues involved. Who owns a patient's MDT and who should have access? What are the correct data protection protocols for a technology that has the potential to integrate a range of patient data and provide a comprehensive view of their health? These are just some of the many issues that will need to be resolved before this technology can enter the mainstream of patient care.

**Adler:** Summary of conceptual, scientific, practical, and ethical challenges and opportunities discussed by other participants in developing medical digital twins.
**An:** Axioms of personalized precision medicine.
**Castiglione:** Constructing a computational representation of the Immune System: necessities, constituents, and operational aspects, along with proposed approaches for model development.
**Eubank:** Lessons to be learned from other fields about data assimilation.
**Glazier:** A theoretical framework for the construction of medical digital twins.
**Helikar:** Towards a General Purpose Immune Digital Twin.
**Jett-Tilton:** Digital twins for PTSD.
**Kirschner:** Models and Tools for building beta versions of digital partners.
**Laubenbacher:** Introduction to the Forum.
**Macklin:** Integration of standardized, reusable descriptions of cell behaviors and interactions.
**Mehrad:** The application of MDTs to the intensive care unit.
**Moore:** Immunologic considerations for building MDT.
**Pasour:** Funding opportunities.
**Shmulevich:** Patient Digital Twin for Acute Myeloid Leukemia.
**Smith:** Immune heterogeneity in the context of lung infection.
**Yankeelov:** Imaging-based digital twins for oncology.
**Ziemssen:** A digital twin for autoimmune diseases accessible to the patient.

Before describing in detail some of the use cases, we briefly sketch several forward-looking scenarios for the potential of MDTs in the context of sepsis as a specific use case. First, it is worth stating explicitly that MDT technology will likely follow the same path as other new technologies. Initial prototypes will have a limited range of capabilities and modest performance and serve perhaps more as a proof-of-concept than fully functional products. The minimum bar any MDT will have to clear, of course, is that it needs to perform at least as well as the standard-of-care for a given application, without any additional risks to patients. The experience gained from initial development and data collected from its use will then drive the development of increasingly more sophisticated versions.

As an example, we present a cascading set of increasingly powerful potential use cases of MDTs in the treatment of sepsis, one of the largest sources of morbidity, mortality and health care costs world-wide (WHO).

1. Early detection of sepsis is a health-monitoring, classification task. This could employ an MDT trained on physiological signals, electronic medical record data and standard laboratory values to deliver an “early warning system” for sepsis.
2. Predicting the trajectory of sepsis. This could be related to the diagnosis task, as certain features might suggest a clinical trajectory that leads to sepsis. It could also be applied to patients already diagnosed with sepsis, to attempt to risk-stratify patients to identify those at risk for clinical deterioration.
3. Optimization of existing therapies for sepsis. The mainstay of current treatment of sepsis involves early administration of antibiotics, source control of potential sources of infection, and physiological support, which includes fluid resuscitation, the use of vasopressors to support blood pressure, and mechanical devices to support failing organs (*i.e*., ventilators and dialysis machines). The combinations of applications, both in time and in degree, could be guided by a sufficiently trained MDT.
4. Discovery and deployment of new therapies. The unfortunate fact of sepsis is that, to date, there is no generally accepted means of interrupting the underlying inflammatory/immune biology that drives sepsis and its subsequent organ failure. Major contributing reasons for this are the overall heterogeneity of the septic population (reflected in a gap between the means of “diagnosing” sepsis and the degree of knowledge regarding the cellular-molecular mechanisms that drive the disease) and the complexity, both in terms of the underlying biological mechanisms and their dynamics in given different insults, of the disease course. In short, effective treatment/control requires identifying the best match between a given patient at a given time with the appropriate set of therapies, and the current means of doing these tasks for a septic patient are woefully inadequate. It is here that MDTs can play an invaluable role in personalizing the characterization of a septic patient so that “right patient, right time, right drug(s)” can be achieved.

## Examples of ongoing mdt projects

We present a selection of ongoing projects by some of the Forum participants. The selection illustrates several disparate types of applications, methodologies, and uses. They are at different stages of development and collectively illustrate the issues we have raised in this meeting report/perspective. A summary of the projects presented at the Forum can be seen in Table 1. Generally, projects can be characterized as follows:

1. Whether the underlying computational model/specification is generated using an existing modeling toolkit/format (which would allow for potentially greater community level expansion) or a “custom” model specific to a particular research laboratory.
2. The disease process addressed by the nascent MDT project.
3. The data types and sources that are available for the data interface between the patient and the digital twin. This ranges from demographic and clinical descriptive data, as found in electronic medical records, the results of medical imaging and tests, and more specific assays that are currently mostly available in the research context (*i.e.*, gene expression, multiplexed mediator assays or highly granular cell type characterization).
4. Whether such a data interface currently exists for the nascent MDT at its current level of development.
5. The modeling method used for the current computational model/specification of the MDT. This includes whether a mechanism-based dynamic model is used, whether a machine learning/artificial intelligence component is part of that dynamic model, or whether the specification is in its early development stages.
6. The approach by which the MDT computational model/specification is personalized (*i.e*., the “twinning” process) to an individual patient in the real world. A precursor to the actual personalization would be the generation of virtual populations, which represents a theoretical distribution of real-world individuals, but have not yet reached a point of development where there can be a direct mapping/connection to an individual patient in the real world.
7. The ostensible clinical goal of the MDT. This could range from diagnosis/surveillance, prognosis/disease trajectory forecasting, optimization and personalization of existing therapies, or the discovery of novel therapies, be they new therapeutic agents, new combinations of existing drugs, or the repurposing of existing drugs into new disease contexts.
8. Whether the MDT project has a patient-facing/engaging interface. This step informs whether, based on the context of its use, such a patient-engagement capability would increase the willingness of potential patients to participate in the MDT project, and helps establish a context for dealing with ethical issues such as patient privacy, data ownership/stewardship and participatory medical decision-making.

**Table 1:** MDT projects in different stages of progress by forum participants. The columns contain information about the technical features of the MDT, the type of data required for calibration, and the application specifications.

Presenter	Modeling framework	Disease	Data structure of the real-world object	Data link b/w in silico & real world	Specification	"Personalization"	Purpose	Patient facing interface
Yankeelov	Custom	Cancer	Multimodal—Molecular, Imaging and clinical data	Yes	Mechanistic PDE/ODE	Yes, from data collected from an intervention	Prognosis, Optimization of existing therapy, control discovery	In development
Shmulevich	Custom	Acute Myeloid Leukemia	Multimodal—Molecular & clinical/epidemiological	Yes	Mechanistic model w/ ML component for feature aggregation	Initialization from a virtual population, collected data from the patient over time	Prognosis, optimize therapy, control discovery	Yes
Kirschner	Custom	Tuberculosis	Molecular, imaging & clinical (animal & patient)	Yes	Mechanistic Model (Multiscale ABM w/ODEs)	Explored possibility space of outcomes & created virtual population	Prognosis, optimization of existing therapies, control discovery of vaccine testing	No, evolving
Smith	Custom	Immune system (Viral infection)	Molecular & clinical	Yes	Mechanistic Model	Virtual populations	Prognosis, scientific discovery	No
Helikar	Custom	Immune system	Molecular & clinical	Evolving	Mechanistic Model	Evolving, can create a VP	Prognosis, Optimization	Planned
Ziemssen	Custom	MS	Molecular, imaging & clinical	Yes	Evolving	Data structure map	Classification, prognosis & optimization	Yes
Castiglione	C-IMMSIM	Immune system	Molecular & clinical	Evolving	Mechanistic Model	Evolving	Prognosis, scientific discovery, control discovery and in silico trials	No
Macklin	Physicell	General purpose	Molecular, cellular, and tissue	Evolving. Recent integration with spatial transcriptomics	Mechanistic Model	Evolving. Agent parameters can be calibrated to individual patient characteristics	Prognosis, optimization & control discovery	No
Glazier	CompuCell3D		Molecular	Evolving	Mechanistic Model	Evolving	Prognosis, optimization & control discovery	No
Jett-Tilton		PTSD	Genomic & clinical	Yes	No, evolving	Yes	Prognosis, optimization & clinical trials	No, evolving
Laubenbacher, Mehrad	Custom	Aspergillosis	Molecular & clinical	Yes	Mechanistic Model (Multiscale ABM w/Boolean networks)	Yes, w/ MLed parameters	Prognosis, optimize therapy, control discovery	No. Doctor facing interface

### A host model for tuberculosis that spans the molecular to the whole host scale (D. Kirschner)

Tuberculosis (TB) imposes a major disease burden on the world, even compared to the COVID pandemic. As much as 25% of the world population is infected with pulmonary *Mycobacterium tuberculosis* (Mtb); however, most patients are classified as having latent tuberculosis (∼90%) with only a small percentage with clinically active disease (∼10%) (WHO). Recent research on both humans and non-human primates has shown that the disease manifests as a range of outcomes (5–8). Individuals with a latent infection sometimes exhibit repeated activation of the pathogen, thus serving as a persistent source of future transmission (9, 10). Key aspects of the human biology underlying the different disease states is still unknown. Understanding what drives different infection outcomes is important as it will inform development and approaches for treatment and prevention.

Pulmonary tuberculosis is characterized by the formation of granulomas, formed as part of the immune response to infection that encapsulate the pathogen. These develop in the lungs of infected hosts after inhalation of mycobacteria (5). It has been shown that a single bacterium can trigger granuloma formation, and there is great diversity among their subsequent development. The structures include bacteria, macrophages, T cells, and other immune cells. While T cells are known to carry out critical functions against Mtb (11), their recruitment to the lungs from the lymph nodes is delayed by as much as several weeks after infection.

We published an organism-scale modeling platform, *HostSim*, of the immune response to *Mycobacterium tuberculosis* consisting of a lymph node compartments, as well as blood and lung compartments (12, 13). With it, one can simulate infection dynamics over long periods of time The model was parameterized and validated with datasets from the literature, including human and non-human primate data. The *HostSim* platform provides the capability to study a range of problems related to the infection and possible intervention strategies.

Recently, we have generated hundreds to thousands of *HostSim* “virtual patients” that are infected with TB at different times and have slightly unique immune characteristics. We refer to this collection of virtual hosts as a “virtual cohort”. This virtual cohort can serve as a bank of digital “partners” that can be closely associated with an actual patient. Initially, a large group of partners (*i.e*., a “digital family”) would be assigned to that patient. Then, as more data become available, the family of partners that are associated with this patient would narrow until a single digital twin remains.

### Virtual patient cohorts for virus infections (A.M. Smith)

Respiratory viral pathogens cause many infections each year, with considerable health and economic burden. Infections with viruses like influenza or SARS-CoV-2 yield a variety of outcomes that range from asymptomatic to fatal. Numerous viral and host factors in addition to complications from other pathogens and underlying diseases can result in heterogeneity in the severity of infection, but their contribution or those from other, hidden mechanisms is unknown. This makes predicting a patient's disease trajectory and the potential for efficacious vaccination or antiviral therapy challenging. The goal of this project is to build virtual patient cohorts (VPCs), with each patient having a personalized immune trajectory (14) to define immunologic processes that initiate diverse outcomes. We construct mechanistic and experimentally validated computational models of the host response and define immune correlates of disease. A focus is on establishing the nonlinearities that drive many immune processes and their connections to disease (15, 16). Within this approach, models are iteratively updated with new data, as immunological knowledge evolves, and as smaller models are validated with targeted experimentation alongside generating diverse VPCs to evaluate underlying comorbidities.

### The digital twin innovation hub (T. Helikar)

The Digital Twin Innovation Hub (17), established in August, 2022, is leading the development of a generic immune digital twin platform that will be contextualizable and applicable to many, and eventually any, immune-related pathology. A comprehensive cellular-level model and map of the immune system, consisting of nearly 30 cell types, over 30 cytokines and immunoglobulins spanning both innate and adaptive immunity has been developed to form a “blueprint” of the general purpose immune digital twin (18). Detailed sub-cellular models of signal transduction and genome-scale metabolism for each of the 30 cell types have also been developed (e.g., dendritic cells, CD4+ T cells (19, 20). Work to integrate these sub-cellular models into a comprehensive multi-scale, multicellular model of the immune system is under way.

Digital Twin Innovation Hub is also developing a software infrastructure to enable the construction, contextualization, personalization, analysis, and simulation of the general purpose immune digital twin. To accomplish this, the Hub is leveraging and building atop of Cell Collective, a web-based collaborative modeling platform (21). To this end, Cell Collective supports several modeling approaches, including logical, kinetic, and constraint-based models, and will soon also support physiologically-based pharmacokinetic/pharmacodynamic models and virtual clinical trials. Cell Collective also provides a repository of computational models, which will provide a gateway to features that will enable their integration into multiscale systems—medical digital twins.

A key principle of Cell Collective is its broad accessibility. To fully leverage the potential of medical digital twins, it will be critical that the technology is accessible to a wide range of user audiences, including translational researchers, clinicians, and patients. As such, in Cell Collective, no mathematical or programming skills are required for users to build, modify, simulate, or analyze models. It also allows users to focus on the mechanistic information used to build and simulate the models rather than dealing with the technicality of formalisms used to build and modify the models.

### C-IMMSIM, a generic immune system simulation platform (F. Castiglione)

The computer model C-IMMSIM can be seen as the outcome of a collaborative effort between a biologist, who provides insights into mechanisms and actions, and a mathematician, who translates that knowledge into a quantitative framework (22). Developing an accurate computer model that represents the complexity of the immune system and produces meaningful outcomes is a challenging task. However, by accepting necessary approximations and building upon solid theoretical mathematical and biological assumptions, along with personalized data to infer the model parameters (23), the C-IMMSIM model can be considered as an underlying generic model of an individual's immune digital twin.

The essential components and prerequisites that have influenced the development of C-IMMSIM are: diversity in specific repertoires; probabilistic actions capturing the inherent stochasticity of many mechanisms; cooperation between different cell types; cell movement and global control; specific cell-cell and cell-molecule interactions; competition and memory cells; clonal selection and proliferation; controls and memory.

All these elements have been incorporated into the C-IMMSIM model using specific mathematical or algorithmic choices. The model can be categorized as an Agent-Based Model (ABM), where individual cells are represented with their unique attributes, such as position, age, membrane receptors, activation status, or differentiation state. ABMs are well-suited for simulating the immune system due to their ability to handle stochastic actions, cell movement, and individual dynamics, while allowing large populations to be simulated and tracked. During the simulation, cells undergo transitions between activation or differentiation states, influenced by stochastic events that rely on the compatibility of their binding sites. While simulating billions of agents and incorporating anatomical variations and an individual's immunological history remains impractical, even with high-performance computers, the overall state of the system in the simulation can still be considered a representative immunological state for an individual. In adopting a digital twin perspective, the model can be customized to align with a patient's physical characteristics and current health status. As a result, it can provide valuable insights into an individual's immune status and potential outcomes in response to specific stimuli. The C-IMMSIM model, in this context, offers a means to simulate a cohort of virtual individuals (referred to as a *virtual cohort*) for the study of specific pathologic and therapeutic conditions, such as type 2 diabetes (24, 25) or COVID-19 vaccination (26).

### Toward a medical digital twin for pneumonia patients in the intensive care unit (R. Laubenbacher, B. Mehrad)

Doctors in intensive care units (ICUs) make decisions in a complex environment, bombarded with thousands of pieces of data, and often under intense time pressure and heterogeneity of patient response to treatment. Available ICU risk calculators provide highly accurate predictions of a patient's length of stay and likelihood of death, but do not provide actionable information about what interventions could be applied to an individual patient to improve the outcome. A common condition of ICU patients is pneumonia. It is the second most common cause of hospital admissions (after admissions for childbirth), with up to 10% of patients requiring an ICU stay. And up to 5% of hospital patients contract pneumonia. It is the main cause of infant mortality. The goal of this project is to build a pneumonia digital twin for ICU patients that serves as a decision support tool for the doctor. Underlying the digital twin is a mathematical model that encodes disease-relevant biological mechanisms and is dynamically calibrated to an individual patient as new data become available. The computational model underlying the pneumonia MDT will be an extension and modification of a model that captures the innate immune response to a fungal infections in the lung, using *Aspergillus fumigatus* as the model pathogen (27).

Ongoing work focuses on an expansion of the model to viral and bacterial pathogens. These studies will be used to expand the computational model for fungal pneumonia, covering all major pathogens causing pneumonia. A tissue culture platform, combined with a cryopreservation technique that keeps human lung tissue functional over several days is being used with lung tissue obtained from surgeries. Collecting heterogenous data from infected tissue from a range of donors allows us to “personalize” the computational model to different donors and investigate heterogeneity in disease progression and response to drugs. This represents the next step in developing the computational model to a state where it can be personalized to actual patients. A part of future work to be done is to integrate this tissue/organ-scale model with a physiological model that allows implementation of all standard treatments available to a pneumonia patient, allowing the comprehensive simulation of patient trajectories under treatment. The final product will be an MDT that is based on a mechanistic computational model, is calibrated dynamically to a pneumonia patient in the ICU for the purpose of helping to plan therapeutic interventions.

### A breast cancer digital twin (T. Yankeelov)

Yankeelov and colleagues have developed mechanism-based mathematical models that are initialized and calibrated with patient-specific, quantitative imaging data for a variety of cancers, especially the breast. The imaging data has included both quantitative positron emission tomography (28) and magnetic resonance imaging (MRI) (29), with a particular emphasis on dynamic contrast enhanced MRI to report on blood flow, and diffusion weighted MRI to report on cellularity. [Using medical imaging data has the advantage of being able to report on anatomical, physiological, cellular, and molecular data non-invasively and at multiple time points to update a digital twin throughout the course of therapy (30)]. Given a high-resolution anatomical image to establish the computational domain, reaction-diffusion equations accounting for tissue mechanical properties and therapeutic regimens are solved over the breast to establish patient specific parameters related to tumor cell migration, tumor proliferation, and response to therapy. After parameterizing the model system, it can be simulated to predict the response of the specific tumor to the specific treatment with high accuracy across space and time (31). It is natural to use the model to form the backbone of an MDT designed to predict and, ultimately, identify therapeutic regimens to optimize the tumor response.

It is important to note that only by employing mechanism-based models can one simulate a range of therapeutic options, including new emerging therapeutics without large clinical trials to use as training data. When using a strictly data-driven approach, one can only search for responses to therapeutic regimens that are included in the training set. By using a mechanism-based model, one is not limited to only the therapeutic regimens included in a historical training set.

### A leukemia digital twin (I. Shmulevich)

The Acute Myeloid Leukemia Digital Twin (AML-DT) project is an initiative supported by the National Cancer Institute (NCI) and the Academy of Finland. It aims to develop a comprehensive digital twin system for AML. This project is characterized by its unique approach to the disease that combines knowledge graphs built by integrating mechanistic models with machine learning approaches applied to data from patients. The models include intracellular gene regulation models and models that capture hematopoiesis and leukemogenesis in a multi-cellular context, incorporating key drivers of tumor progression. The overarching goal of this project is to predict disease progression and optimize response to therapies, thereby revolutionizing the way we understand and treat AML. The project is a collaborative effort, bringing together diverse technologies, such as modeling, machine learning, human-computer interaction, and clinical practice.

The development of the AML digital twin necessitates several types of data that capture different aspects of a patient, such as clinical data, cytogenetics, mutation panels, and flow cytometry measurements. These data are utilized to individualize each digital twin, creating a personalized representation of the patient's disease state. Alongside patient-specific data, the project also incorporates public datasets for the construction of knowledge graphs. These datasets include molecular profiling, as well as *ex vivo* drug sensitivity data, both linked with clinical outcomes. The integration of individual patient data and public datasets enhances the digital twin's ability to predict disease progression and drug response, which is the primary objective of this project.

We have captures the central features of the immune system that contribute to pathogenesis of AML through the integration of detailed domain-specific knowledge graphs with multiscale dynamical models of the tumor microenvironment. These models incorporate key mechanisms of cancer progression, which can aid in the development of new therapies.

The digital twin approach goes beyond being just a model. Each AML patient will have a digital twin individually tailored using information produced in a clinical laboratory. This is combined with a model-based approach for making personalized predictions. An important aspect of this approach is the learning-cycle, where patient outcomes are continuously utilized to improve predictions. Over time, the system will improve as discoveries are made related to the biological aspects that are most important for accurate prediction of patient outcomes. This approach allows us to capture the temporal evolution of the disease state for a particular patient, providing a more accurate and personalized prediction of disease progression and treatment response.

## Conclusion

We are in the very early stages of developing MDTs as a key technology for personalized medicine. To make an analogy to the development of aviation, we are only a short time past the first flight at Kitty Hawk in the early years of last century. While it would a century until we can travel between continents in record time and comfort, it is worth noting that only a decade after the Wright Brothers' first flight, all major combatants in World War I fielded an air force. Likewise, while it might take a long time still until MDTs are in standard use across medicine, this technology might provide a measurable improvement over our current capabilities. To achieve this, we must collectively engage in a focused effort on improving our understanding of human biology and translate it into computational models, enable and implement the collection of human data and algorithms needed to personalize these models, and adapt them for practical use in healthcare. While this will be very challenging, it is necessary for a new, improved, paradigm in human health. The sample projects we described are, for the most part, far away from being functional and useful MDTs. All of them, however, represent progress toward applications and technology platforms for truly personalized medicine.
